# Cancer-Testis Antigen Expression in Serous Endometrial Cancer with Loss of X Chromosome Inactivation

**DOI:** 10.1371/journal.pone.0137476

**Published:** 2015-09-11

**Authors:** Jun Kang, Hee Jin Lee, Sun-Young Jun, Eun Su Park, Lee-so Maeng

**Affiliations:** 1 Department of Hospital Pathology, Incheon St. Mary's Hospital, College of Medicine, The Catholic University of Korea, Inchun, Republic of Korea; 2 Department of Pathology, University of Ulsan College of Medicine, Asan Medical Center, Seoul, Republic of Korea; IRCCS National Cancer Institute, ITALY

## Abstract

**Background:**

Cancer-testis antigens (CTAs) are potential targets for cancer immunotherapy. Many CTAs are located on the X chromosome and are epigenetically regulated. Loss of X chromosome inactivation (XCI) is observed in breast and ovarian cancers and is thought to be related to the overexpression of CTAs. We investigated the relation between expression of CTAs and loss of XCI in endometrial cancer.

**Materials and Methods:**

We used data generated by The Cancer Genome Atlas Genome Data Analysis Centers and data for *Xist* knockout mice available at the Gene Expression Omnibus.

**Results:**

The status of XCI was estimated by methylation status, and deletion or gain of the X chromosome. The endometrial cancers were classified into the following three groups: preserved inactivated X chromosome (Xi) (n = 281), partial reactivation of Xi (n = 52), and two copies of active X group (n = 38). Loss of XCI was more common in serous adenocarcinoma. Expression of CTAs increased in endometrial cancer with loss of XCI, which was accompanied by global hypomethylation. Expression of CTAs did not increase in *Xist* knockout mice.

**Conclusions:**

Loss of XCI is common in serous adenocarcinoma. Global hypomethylation, and not loss of XCI, is the main mechanism of overexpression of CTAs.

## Introduction

Cancer is immunogenic. Cancer cells aberrantly express antigenic proteins that are usually not expressed in normal cells. These antigenic proteins induce cancer immunity. Cancer immunotherapy enhances cancer immunity [1, 2]. Cancer-testis antigens (CTAs) are potential targets for cancer immunotherapy [3, 4]. CTAs are antigenic because they are not expressed in normal cells, except in germ cells of the testis [5–10]. Many CTAs are located on the X chromosome and are epigenetically regulated [11]. Global hypomethylation of cancer cells causes the aberrant expression of CTAs in cancer cells [12, 13].

Female have two copies of the X chromosomes, whereas male have one. To compensate X chromosome dosage, one of the two copies of the X chromosomes is randomly inactivated during a process called X chromosome inactivation (XCI) in female. XCI is initiated and maintained by X-inactive specific transcript (XIST), a 17 kilobase noncoding RNA. *XIST* transcript coats the inactive X chromosomes (Xi). The loss of XCI occurs in ovarian and breast cancers [14–17]. It is thought that this loss increases X chromosome dosage and expression of oncogenes located on the X chromosome [18]. In a previous study, we demonstrated that loss of XCI increased expression of CTAs located on the X chromosome (XCTAs), including X antigen family member 3 (*XAGE3*) and melanoma antigen family A4 (*MAGEA4*). This result suggests that expression of CTAs is regulated by not only global hypomethylation but also by loss of XCI [15].

Loss of XCI occurs in high-grade ovarian serous adenocarcinoma and basal-like breast cancer [19]. Serous adenocarcinoma of endometrium shares many clinicopathologic features and genomic alterations with ovarian and basal-like breast cancers. Endometrial cancer is classified into endometrioid adenocarcinoma and serous adenocarcinoma [20]. Endometrioid adenocarcinoma is associated with estrogen excess and endometrial polyp. Serous adenocarcinoma occurs in older patients and is frequently associated with TP53 mutation and high degree of chromosome copy number variations (CNV) [19].

In this study, we determined whether loss of XCI occurs in endometrial cancer of the serous type and whether this loss results in increased XCTA expression.

## Materials and Methods

### The Cancer Genome Atlas (TCGA) data

We used open source data generated by TCGA genome data analysis centers [19]. Data on methylation 450K were downloaded from the TCGA Data Portal (https://tcga-data.nci.nih.gov/tcga/tcgaHome2.jsp). Data on CNV and RNA expression were downloaded from the Broad genome data analysis center Firehose website (http://gdac.broadinstitute.org/). Data on RNA expression of *XIST* and clinical data were gathered using the CGDS-R package, which is a package of R for querying the Cancer Genomics Data Server and is hosted by the Computational Biology Center at Memorial-Sloan-Kettering Cancer Center [21]. The beta values for DNA methylation status were estimated using the Illumina Infinium Human Methylation 450K arrays. The beta value was calculated as an estimate of the ratio of intensities between methylated and unmethylated alleles. Segmented copy number was estimated by log2 of the ratio of total intensity of the tumor and the normal tissue using Affymetrix SNP6.0. Normalized RNA-Seq by expectation maximization (RSEM) were used as an estimate for mRNA expression [22]. Detailed information about the patients and experiment methods have been described elsewhere [19].

### Gene Expression Omnibus data

We analyzed gene expression profile data of *Xist* deleted blood cells, including B-lymphoid, myeloid, erythroid cells, and hematopoietic stem cells, of mice using the Cre/LoxP system. The data were downloaded from the Gene Expression Omnibus website (http://www.ncbi.nlm.nih.gov/sites/GDSbrowser?acc=GDS4755) [18]. The expression data were generated from Affymetrix mouse Gene 1.0ST arrays (Affimetrix). Detailed experimental methods have been described elsewhere [18].

### Ethics statement

Tissue used by TCGA was collected after obtaining approval from local institutional review boards (http://cancergenome.nih.gov/abouttcga/policies/informedconsent). The authors of the animal study for tissue specific *Xist* deletion had obtained approval from the Institutional Animal Care and Use Committee [18].

### TCGA molecular subtype

TCGA research network classified endometrial cancer into four TCGA molecular subtypes including *POLE* (ultramutated), MSI (hypermutated), copy-number low (endometrioid) and copy-number high (serous-like) [19]. *POLE* subtype was endometrial cancer with high mutation frequency and having *POLE* exonuclease mutations. MSI subtype was endometrial cancer with high mutation frequency and microsatellite instability (MSI) high. Copy-number low subtype was endometrial cancer with low degree of CNV and endometrioid histological type. Copy-number high subtype was endometrial cancer with high degree of CNV and serous histological type.

### Clustering analysis of X chromosome methylation

We selected 5441 methylation array probes that target CpG islands on the X chromosome. CpG islands are major sites of methylation during XCI. Beta values of selected probes were analyzed using hierarchical clustering with complete agglomeration. The clustering was done separately for each serous and endometrial histological type. Expression of *XIST* and loss of the p or q arm of the X chromosome were evaluated to assess the status of X chromosome inactivation.

### Expression of CTAs

We selected 48 XCTAs and eight CTAs located on somatic chromosomes for analysis of TCGA data. Mean RNA expression of the selected CTAs was compared according to TCGA molecular subtype, histological type, and status of XCI using *t*-test or analysis of variance (ANOVA) test. We selected 16 XCTAs (20 array probes) for analysis of the *Xist* knockout mice. Mean RNA expression was compared by strip chart analysis. Statistical analysis was not performed because of the small sample size. The names of selected CTAs were listed (S1 Text).

### Methylation of somatic chromosome for assess global hypomethylation

We selected 239,905 methylation array probes that target CpG islands on somatic chromosomes. Mean beta values of selected probes were compared according to TCGA molecular subtype, histological type, and status of XCI using *t*-test or ANOVA test.

### Clinical data and survival

Patient characteristics and tumor characteristics were compared according to the status of XCI. Significance was estimated using Fisher’s exact test for categorical variables and ANOVA test for continuous variables. Univariate survival analysis was performed using the log-rank test. Multivariate survival analysis was performed using Cox regression tests to adjust for clinical stage.

## Results

### Clustering of X chromosome methylation

**Fig 1:**
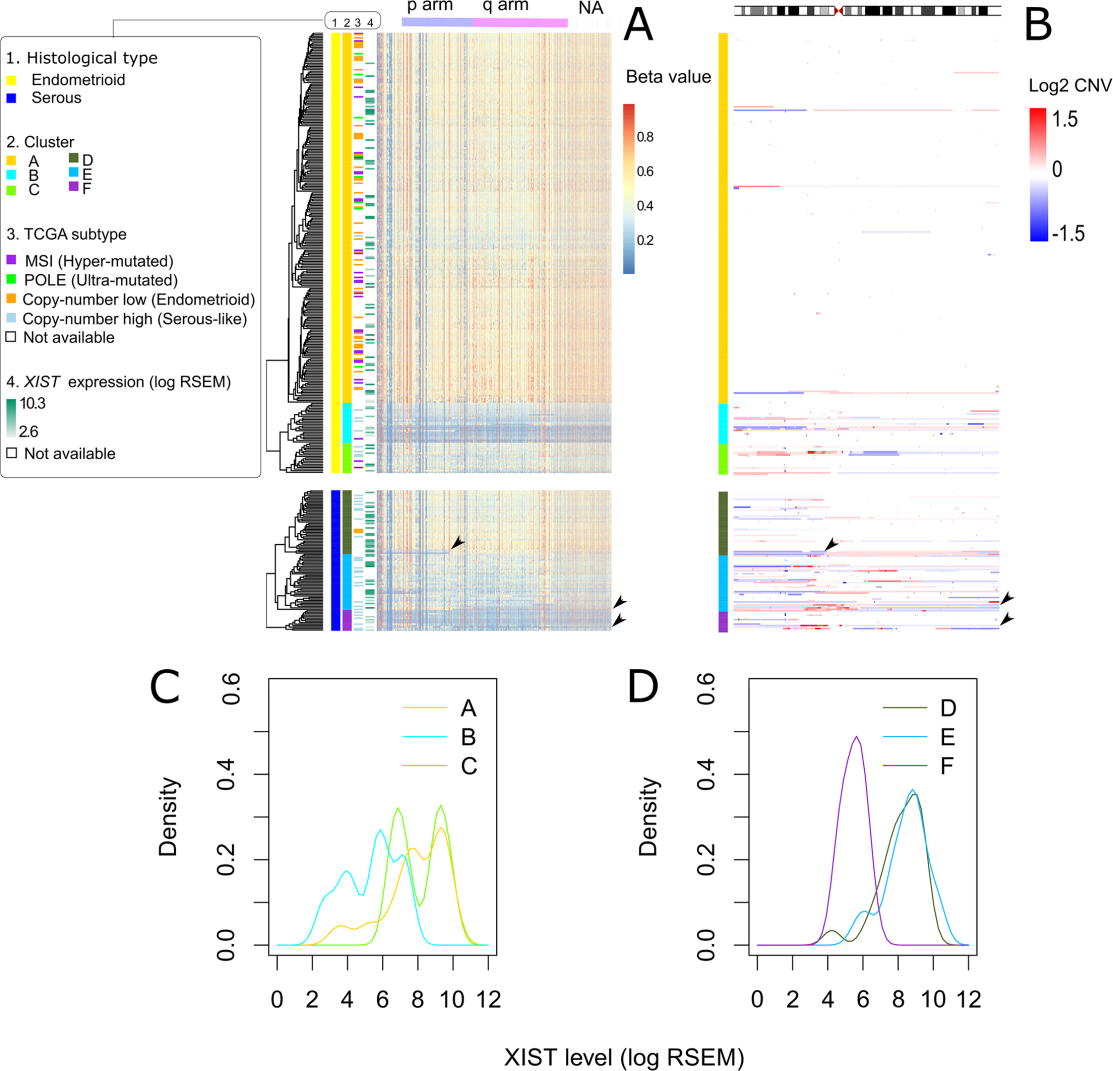
Status of X chromosome inactivation. A) Clustering of 371 endometrial carcinomas with beta value of methylation at CpG islands on the X chromosome. Clustering analyses were performed separately for serous and endometrioid adenocarcinomas. Column shows methylation array probes arranged by X chromosome location. The row shows TCGA cases ordered by cluster group. The left side-columns indicate the histological type, cluster, TCGA subtype and *XIST* expression. Most cases of serous histological type are copy-number high (serous-like) TCGA subtype. Cases of endometrioid histological type are mixed with four TCGA subtypes. B) Colors represent segmental copy number variation of the X chromosome of endometrial carcinoma. The row shows TCGA cases arranged to correspond with methylation clusters. C, D) Density lines show distribution of *XIST* expression level in each cluster. Clusters are distinguished by different colors. Scale of X-axis is log transformed RSEM. A, B) Arrow heads indicate arm-level deletion of inactive X chromosome.

We included two histological types (endometrioid and serous) in this study. We classified 276 endometrioid and 95 serous endometrial carcinomas into six clusters according to X chromosome methylation (Fig 1A). The status of XCI of clusters was characterized with *XIST* expression and arm-level X chromosome deletion. Data on *XIST* expression was not available in 253 (68.2%) cases. Clusters A to C were endometrioid adenocarcinomas and Clusters D to F were serous adenocarcinoma. Clusters A and D showed hypermethylation of most of the loci included on the X chromosome (Fig 1A) and high *XIST* expression (Fig 1C and 1D). These results suggest that Clusters A and D had both active (Xa) and Xi. Clusters B and F showed hypomethylation at most loci on the X chromosome (Fig 1A) and low *XIST* expression (Fig 1C and 1D). Both hypomethylation of the entire X chromosome and low *XIST* expression indicated absence of Xi. The absence of Xi could be either the result of one Xa with the accompanying deletion of a whole Xi, or the result of multiple copies of Xa without Xi due to a segregation error. Because deletion of a whole Xi did not increase dosage of X chromosome, we determined whether the absence of Xi was accompanied with deletion of whole Xi or not. Whole X chromosome deletion was not observed in Clusters B or F (Fig 1B). These results suggested that Clusters B and F had two copies of Xa. Clusters C and E showed high *XIST* expression (Fig 1C and 1D), like Clusters A and D, but were only partially hypomethylated (Fig 1A). The high *XIST* expression suggested the presence of Xi. The partial hypomethylation suggested that the Xi of Clusters C and E were partially reactivated. Selective arm-level hypomethylation of X chromosome was observed at p arm (the last three cases of Cluster D in Fig 1A) and q arm (the last five cases of Cluster E and the third last case of Cluster F in Fig 1A). These selective arm-level hypomethylation corresponded to arm-level deletions of the X chromosome (Fig 1B). The selective arm-level hypomethylation suggested that the deletions occurred in the Xi, but not in Xa. The endometrial cancers were classified into the following three groups: 1) “preserved Xi” in the cases of Clusters A and D and in cases with q arm loss of Xi of Clusters E and F; 2) “partial reactivation of the Xi” (Xa^+^) for Clusters C and E; and 3) “two copies of the Xa” (two Xa) for Clusters B and F.

### Expression of CTAs and loss of XCI

**Fig 2:**
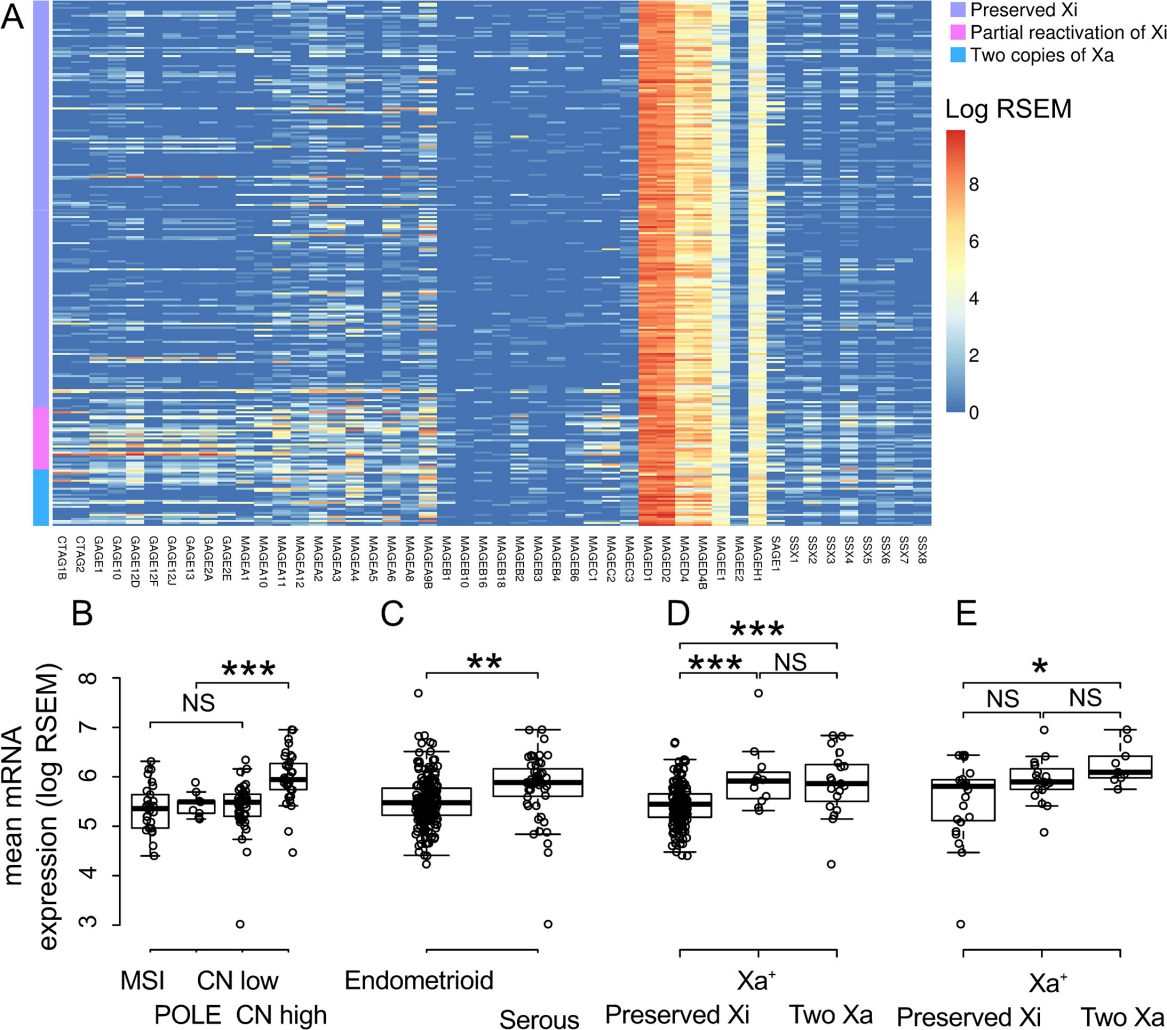
RNA expression of cancer-testis antigens located on the X chromosome. A) The color of the cells represent RNA expression level (log transformed RSEM). The column shows cancer-testis antigens located on the X chromosome. The row shows cases ordered by cluster group. The left side-column indicates the group of methylation clustering. B-D) Box plots represent the distribution of mean RNA expression of cancer-testis antigens located on the X chromosome (log transformed RSEM). The top and bottom of the box are the 75th and 25th percentiles, respectively, and the line in the box is the median. Expression of cancer-testis antigens is compared according to status of X chromosome inactivation in endometrioid histological subset (C) and serous histological subset (D). NS: not significant, *: *P* < 0.05, **: *P* < 0.01, *** *P* < 0.001

**Fig 3:**
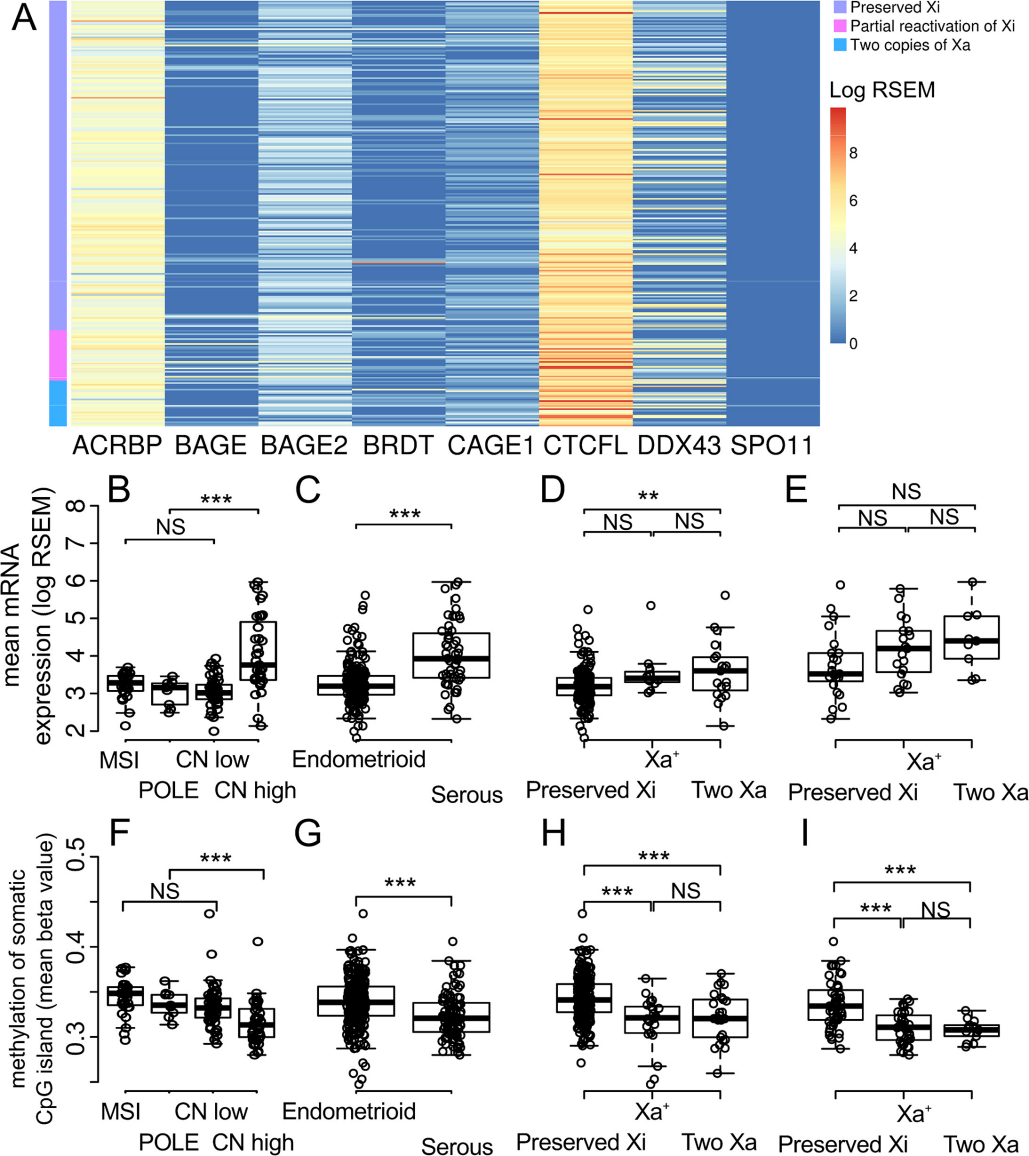
RNA expression of cancer-testis antigens located on somatic chromosomes and global methylation. A) The color of the cells represent RNA expression level (log transformed RSEM). The column shows cancer-testis antigens located at somatic chromosome. The row shows cases ordered by cluster group. The left side-column indicates the group of methylation clustering. B-D) Box plots represent the distribution of mean RNA expression of cancer-testis antigens located on somatic chromosomes (log transformed RSEM). The top and bottom of the box are the 75th and 25th percentiles, respectively, and the line in the box is the median. D) Expression of Cancer-testis antigens located at somatic chromosome is compared according to status of X chromosome inactivation in endometrioid histological subset (C) and serous histological subset (D). E-G) Box plots represent the distribution of mean methylation of CpG islands in somatic chromosomes (beta value). The top and bottom of the box are the 75th and 25th percentiles, respectively, and the line in the box is the median. G) Mean methylation of CpG islands in somatic chromosomes (beta value) is compared according to status of X chromosome inactivation in endometrioid histological subset (C) and serous histological subset (D). NS: not significant, *: *P* < 0.05, **: *P* < 0.01, *** *P* < 0.001

Of the 371 cases, 110 cases (29.6%) were excluded in the analysis of expression of CTAs, due to the absence of expression data. The expression level was varied among CTAs (Fig 2A and Fig 3A). Most CTAs were rarely expressed and the aberrant expression was enriched in Xa^+^ and Two Xa. However some CTAs were highly expressed in most cases and over-expressed in Xa^+^ and Two Xa; those CTAs were MAGED gene family, *Melanoma Antigen Family H*, 1 (*MAGEH1*), *Acrosin Binding Protein* (*ACRBP*) and *CCCTC-Binding Factor (Zinc Finger Protein)-Like* (*CTCFL)*.

XCTAs were more highly expressed in copy number high (serous-like) TCGA molecular subtype and serous histological type than other TCGA molecular subtype and endometrioid histological type. Xa^+^ and two Xa showed higher expression of XCTAs than preserved Xi in both endometrioid and serous histological types. However the *P* value was not significant between Xa^+^ and preserved Xi in serous histological type (Fig 2).

Expression of CTAs located on somatic chromosomes was similar with that of XCTAs. It was higher in copy number high (serous-like) TCGA molecular subtype and serous histological type than other TCGA molecular subtype and endometrioid histological type. Xa^+^ and two Xa showed higher expression of CTAs located on somatic chromosomes than preserved Xi in both endometrioid and serous histological types. The *P* value was only significant between Two Xa and preserved Xi in endometrioid histological type but *P* value among others were not significant (Fig 3).

### Global hypomethylation

The mean beta value of the CpG islands of somatic chromosomes was lower in copy-number high (serous-like) TCGA molecular subtype, serous histological type, Xa^+^ and two Xa than other TCGA molecular subtype, endometrioid histological type and preserved Xi (Fig 3F–3I).

### Expression of CTAs in *Xist* knockout mice

**Fig 4:**
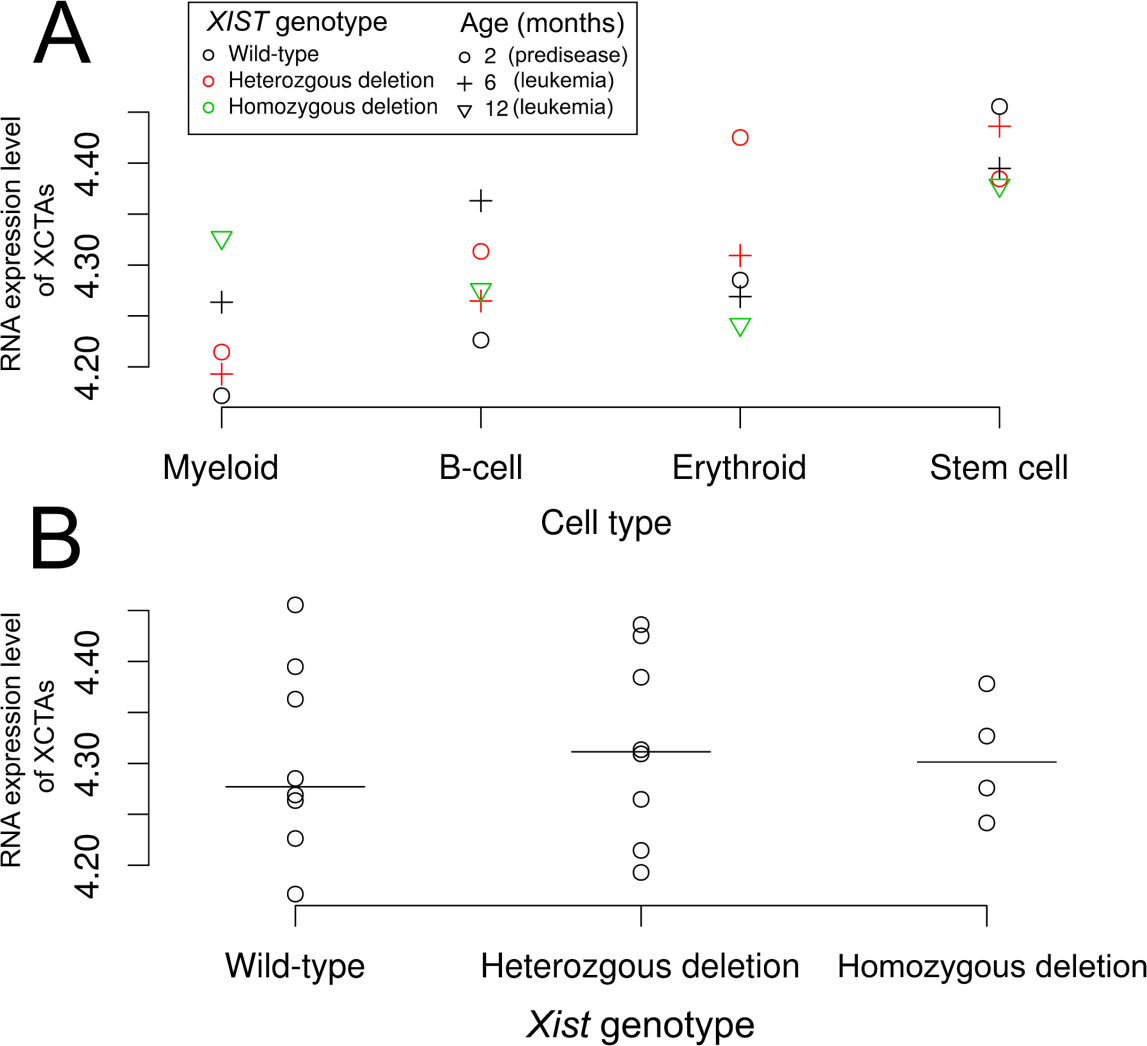
RNA expression of cancer-testis antigens located on the X chromosome of *Xist* knockout mice. The points represent mean RNA expression of 16 cancer-testis antigens located on the X chromosome among blood cell types (A) and *Xist* genotypes (B). Bars represent medians (B).

Expression of XCTAs was higher in hematopoietic stem cells than in other blood cells, including myeloid, B-cell, and erythroid cells (Fig 4A). The mean expression of XCTAs was not significantly different among different *Xist* genotypes, including heterozygotic deletion, homozygotic deletion, and wild-type (Fig 4B).

### Clinical and tumor characteristics

Xa^+^ and two Xa were more frequent in serous histological type and copy-number high (serous like) TCGA molecular subtype. Furthermore Xa^+^ and two Xa were mostly detected in copy-number high (serous like) TCGA molecular subtype (Table 1). Xa^+^ and two Xa patients were older and in menopausal state than preserved Xi patients (Table 2). There were no statistically significant differences in race, ethnicity, clinical stage, or residual tumor status among the groups according to the status of Xi.

**Table 1:** Distribution of status of XCI among histological types and TCGA subtypes.

	Histological type (*N* = 371)			TCGA Subtype (*N* = 122)*				
	Endometrioid	Serous	*P*	Copy-number high (Serous-like)	Copy-number low (Endometriod)	MSI (Hyper-mutated)	POLE (Ultra-mutated)	*P*
Preserved Xi	232 (84.1%)	49 (51.6%)	<0.001	11 (31.4%)	49 (100%)	26 (89.7%)	9 (100%)	<0.001
Xa^+^	19 (6.9%)	33 (34.7%)		14 (40%)	0 (0%)	2 (6.9%)	0 (0%)	
Two Xa	25 (9.1%)	13 (13.7%)		10 (28.6%)	0 (0%)	1 (3.4%)	0 (0%)	

Xa^+^: Partial reactivation of Xi, Two Xa: Two copies of Xa

* Data on TCGA subtype was not available in 249 (67.1%) cases.

**Table 2:** Patient and tumor characteristics according to status of X chromosome inactivation.

	All (n = 371)	Preserved Xi (n = 281)	Xa^+^ (n = 52)	Two Xa (n = 38)	*P*
Age (*N* = 371)	64.0 (11.3)	62.2 (11.4)	69.8 (9.1)	68.9 (8.8)	<0.001^e^
Ethnicity (*N* = 279) ^a^					0.215
Hispanic or Latino	9 (3.2%)	5 (2.4%)	2 (5.3%)	2 (6.5%)	
Not Hispanic or Latino	270 (96.8%)	205 (97.6%)	36 (94.7%)	29 (93.5%)	
Race (*N* = 350) ^a^					0.527
White	271 (77.4%)	208 (78.2%)	35 (72.9%)	28 (77.8%)	
Black or African American	63 (18.0%)	44 (16.5%)	11 (22.9%)	8 (22.2%)	
Asian^b^	16 (4.6%)	14 (5.3%)	2 (4.2%)	0 (0.0%)	
Menopause status^a^ ^,^ ^c^ (*N* = 335)					0.024^d^
Post	293 (87.5%)	212 (84.1%)	49 (98.0%)	32 (97.0%)	
Pre	26 (7.8%)	25 (9.9%)	1 (2.0%)	0 (0.0%)	
Peri	16 (4.8%)	15 (6.0%)	0 (0.0%)	1 (3.0%)	
Residual tumor^a^ ^,^ ^d^ (*N* = 305)					0.674
R0	248 (81.3%)	186 (82.3%)	36 (78.3%)	26 (78.8%)	
R1	18 (5.9%)	12 (5.3%)	2 (4.3%)	4 (12.1%)	
R2	13 (4.3%)	9 (4.0%)	3 (6.5%)	1 (3.0%)	
RX	26 (8.5%)	19 (8.4%)	5 (10.9%)	2 (6.1%)	
Clinical stage (*N* = 370) ^a^					0.29
Stage I-II	267 (72.2%)	209 (74.4%)	34 (66.7%)	24 (63.2%)	
Stage III	82 (22.2%)	57 (20.3%)	15 (29.4%)	10 (26.3%)	
Stage IV	21 (5.7%)	15 (5.3%)	2 (3.9%)	4 (10.5%)	
*XIST* expression (log RSEM) (*N* = 118) ^a^	7.7 (1.7)	7.9 (1.7)	8.3 (1.2)	5.4 (1.3)	<0.001^e^

^a^ Number of cases with available

^b^ Asian includes Asian, Native American, Native Alaskan, Native Hawaiian and other Pacific Islanders

^c^ post: prior bilateral ovariectomy or >12 mo since last menstrual period with no prior hysterectomy, pre: <6 months since last menstrual period and no prior bilateral ovariectomy and not on estrogen replacement, peri: 6–12 months since last menstrual period

^d^ R0: no residual tumor, R1: microscopic residual tumor, R2: macroscopic tumor, RX: presence of residual tumor cannot be assessed

^e^ Statistically significant

Xa^+^: Partial reactivation of Xi, Two Xa: Two copies of Xa

### Prognostic significance

**Fig 5:**
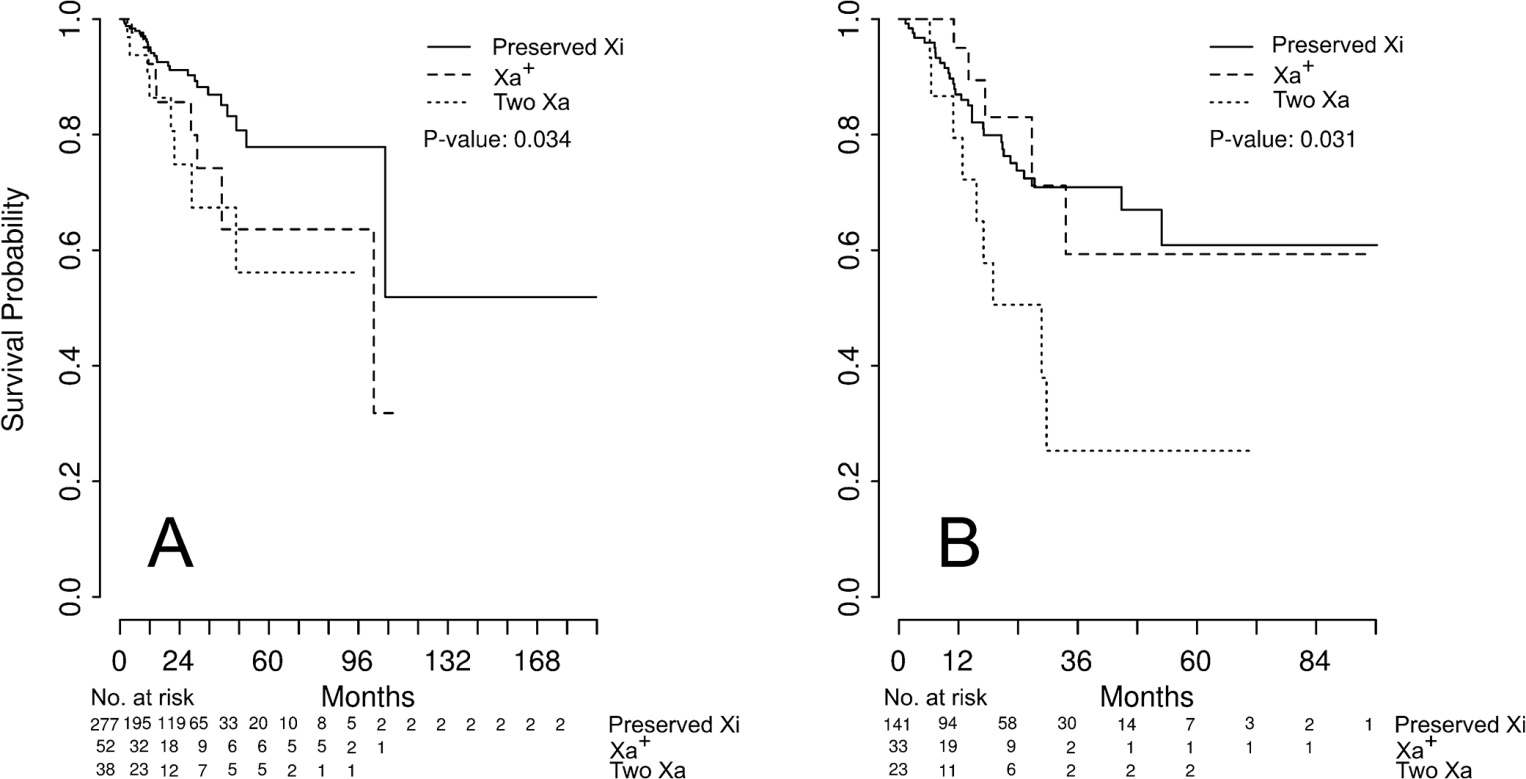
Prognosis in patients showing dysregulation of X chromosome inactivation. Overall (A) and disease-free survival (B) were compared according to the status of X chromosome inactivation. Significance was estimated by the overall log-rank test.

Out of the 371 patients, five (1.3%) and 174 (46.9%) were excluded from the overall and disease free survival analyses, respectively, due to the absence of available data. The two Xa patients showed worse overall and disease-free survival in univariate survival analysis (Fig 5A). The Xa^+^ patients showed the worse overall survival, but not disease-free survival (Fig 5B). After adjustment for clinical stage, the difference among the groups according to the status of Xi was not significant (Table 3). The hazard ratio for adverse clinical outcomes of loss of XCI was approximately 1.6, with a wide confidential internal (0.7–3.5). In sub-analysis for each histological type, Two Xa showed worse overall survival in endometrioid histological type and worse disease-free survival in serous histological type; however *P*-value was not significant (S1 Fig). After adjustment for clinical stage, two Xa showed significantly worse disease-free survival in serous histological type (S1 Table).

**Table 3:** Overall and disease free survival data using multivariate Cox regression test.

	Overall survival		Disease free survival	
	HR (95%CI)	*P*	HR	*P*
Stage I-II	Reference	Reference	Reference	Reference
Stage III	4.24 (2.14–8.4)	<0.001	2.13 (1.03–4.4)	0.041
Stage IV	7.31 (3.23–16.58)	<0.001	6.66 (3.18–13.92)	<0.001
Preserved Xi	Reference	Reference	Reference	Reference
Xa^+^	1.65 (0.77–3.52)	0.198	0.71 (0.27–1.84)	0.478
Two Xa	1.58 (0.7–3.58)	0.271	1.58 (0.73–3.42)	0.25

HR: hazard ratio, CI: confidential interval, Xa^+^: Partial reactivation of Xi, Two Xa: Two copies of Xa

## Discussion

Loss of XCI can be induced by segregation error, causing daughter cancer cells to inherit two uniparental Xa (two Xa) instead of one Xa and one Xi [23]. Another mechanism for loss of XCI is demethylation of Xi (Xa^+^) associated with global hypomethylation. We previously reported that both segregation error and demethylation of Xi occurs in high grade ovarian serous adenocarcinoma [15]. In this study, we tried to determine whether loss of XCI also occurs in serous adenocarcinoma of the endometrium. We found that loss of XCI almost exclusively occurred in serous adenocarcinoma of the endometrium or serous-like TCGA molecular subtype. As in ovarian cancer, loss of XCI is caused by both segregation error and demethylation of Xi in endometrial cancer. The exclusive occurrence of loss of XCI in serous adenocarcinomas of the endometrium and ovary is well correlated with the high degree of CNV in those carcinomas. This suggests that loss of XCI can occur in other cancers with a high degree of CNV, such as colorectal cancer, non-small cell lung cancer, and head and neck squamous cell carcinoma [24–27].

In our previous study, some XCTAs, including *XAGE3* and *MAGEA4*, were overexpressed in ovarian cancer with a loss of XCI [15]. We want to know whether CTAs are also overexpressed in endometrial cancer with loss of XCI. In this study, overall expression of XCTAs increased in endometrial cancer with loss of XCI, in both two Xa and Xa^+^. Furthermore, both Xa^+^ and two Xa showed global hypomethylation and overexpression of CTAs located at somatic chromosomes. In our previous study, the two Xa of ovarian cancer was globally hypomethylated, like that of endometrial cancer [15]. It was uncertain that the effect on expression of XCTAs of loss of XCI is independent from global hypomethylation. We analyzed expression of XCTAs in *Xist* knockout mice to determine if loss of Xi increases expression of XCTAs independently of global hypomethylation. Expression of XCTAs did not increase in *Xist* knockout mice. We conclude that loss of XCI has a much smaller effect on expression of XCTAs than global hypomethylation. The overexpression of XCTAs in cancer with loss of XCI may be primarily caused by the accompanying global hypomethylation. However the effect on expression of XCTAs of loss of XCI cannot be generalized in other malignancies. Furthermore the result of *Xist* knockout mice has limitation to apply human solid organ malignancies. Further study is required to generalize the conclusion.

Loss of XCI is oncogenic in mice, which suggests that loss of XCI could have a role in tumor progression or poor patient prognosis. The hazard ratio for adverse clinical outcomes of loss of XCI was approximately 1.6 with wide a confidential internal (0.7–3.5) which is similar to that of ovarian cancer [15]. The disease-free survival result was not consistent with the overall survival. This discrepancy might be caused by bias due to a large proportion of missing data. The trend of worse prognosis of two Xa was observed in sub-analysis of endometrioid and serous histological subsets. Collectively, our results suggest that loss of XCI is associated with a slightly higher risk for adverse clinical outcomes but with limited evidence.

## Conclusion

Our observations provide evidence that loss of XCI is common in serous adenocarcinoma of the endometrium and in serous-like adenocarcinoma, which showed a high degree of CNV. This result suggests that loss of XCI occurs not only in serous ovarian cancer or basal-like breast cancer but also in cancer with high degree of CNV. We found that global hypomethylation, and not loss of XCI, was the main mechanism of overexpression of XCTAs, and that loss of XCI has a slightly higher risk for adverse clinical outcome but with limited evidence.

## Supporting Information

S1 Text List of selected cancer-testis antigens. (DOCX)

S1 Fig Sub-analysis for survival of each histological type. Significance was estimated by the overall log-rank test.
(TIF)

S1 Table Sub-analysis for multivariate Cox regression test of each histological type. (DOCX)
