# Supplementary material for: Cancer-Testis Antigen Expression in Serous Endometrial Cancer with Loss of X Chromosome Inactivation
Source: PLoS One. 2015 Sep 11;10(9):e0137476. doi: 10.1371/journal.pone.0137476 (PMC4567132; doi:10.1371/journal.pone.0137476)
Supplement: S1 Text — (DOCX) [file pone.0137476.s001.docx]

List of cancer testis antigens located at X chromosome used in analysis

CTAG1B: Cancer/Testis Antigen 1B

CTAG2: Cancer/Testis Antigen 2

GAGE1: G Antigen 1

GAGE10: G Antigen 10

GAGE12D: G Antigen 12D

GAGE12F: G Antigen 12F

GAGE12J: G Antigen 12J

GAGE13: G Antigen 13

GAGE2A: G Antigen 2A

GAGE2E: G Antigen 2E

MAGEA1: Melanoma Antigen Family A, 1

MAGEA10: Melanoma Antigen Family A, 10

MAGEA11: Melanoma Antigen Family A, 11

MAGEA12: Melanoma Antigen Family A, 12

MAGEA2: Melanoma Antigen Family A, 2

MAGEA3: Melanoma Antigen Family A, 3

MAGEA4: Melanoma Antigen Family A, 4

MAGEA5: Melanoma Antigen Family A, 5

MAGEA6: Melanoma Antigen Family A, 6

MAGEA8: Melanoma Antigen Family A, 8

MAGEA9B: Melanoma Antigen Family A, 9B

MAGEB1: Melanoma Antigen Family B, 1

MAGEB10: Melanoma Antigen Family B, 10

MAGEB16: Melanoma Antigen Family B, 16

MAGEB18: Melanoma Antigen Family B, 18

MAGEB2: Melanoma Antigen Family B, 2

MAGEB3: Melanoma Antigen Family B, 3

MAGEB4: Melanoma Antigen Family B, 4

MAGEB6: Melanoma Antigen Family B, 6

MAGEC1: Melanoma Antigen Family C, 1

MAGEC2: Melanoma Antigen Family C, 2

MAGEC3: Melanoma Antigen Family C, 3

MAGED1: Melanoma Antigen Family D, 1

MAGED2: Melanoma Antigen Family D, 2

MAGED4: Melanoma Antigen Family D, 4

MAGED4B: Melanoma Antigen Family D, 4B

MAGEE1: Melanoma Antigen Family E, 1

MAGEE2: Melanoma Antigen Family E, 2

MAGEH1: Melanoma Antigen Family H, 1

SAGE1: Sarcoma Antigen 1

SSX1: Synovial Sarcoma, X Breakpoint 1

SSX2: Synovial Sarcoma, X Breakpoint 2

SSX3: Synovial Sarcoma, X Breakpoint 3

SSX4: Synovial Sarcoma, X Breakpoint 4

SSX5: Synovial Sarcoma, X Breakpoint 5

SSX6: Synovial Sarcoma, X Breakpoint 6

SSX7: Synovial Sarcoma, X Breakpoint 7

SSX8: Synovial Sarcoma, X Breakpoint 8

List of cancer testis antigens located at somatic chromosomes used in analysis

ACRBP: Acrosin Binding Protein

BAGE: B Melanoma Antigen

BAGE2: B Melanoma Antigen Family, Member 2

BRDT: Bromodomain, Testis-Specific

CAGE1: Cancer Antigen 1

CTCFL: CCCTC-Binding Factor (Zinc Finger Protein)-Like

DDX43: DEAD (Asp-Glu-Ala-Asp) Box Polypeptide 43

SPO11: **SPO11** Meiotic Protein Covalently Bound To DSB

List of cancer testis antigens located at X chromosomes of mouse used in analysis

Ctag2: cancer/testis antigen 2

Magea10: melanoma antigen family A, 10

Magea3: melanoma antigen family A, 3

Magea4: melanoma antigen family A, 4

Magea5: melanoma antigen family A, 5

Magea6: melanoma antigen family A, 6

Magea8: melanoma antigen family A, 8

Magea18: melanoma antigen family B, 18

Mageb3: melanoma antigen family B, 3

Mageb4: melanoma antigen family B, 4

Mageb5: melanoma antigen family B, 5

Maged1: melanoma antigen family D, 1

Maged2: melanoma antigen family D, 2

Magee1: melanoma antigen family E, 1

Magee2: melanoma antigen family E, 2

Mageh1: melanoma antigen family H, 1
