# Supplementary figures and images for: Cancer-Testis Antigen Expression in Serous Endometrial Cancer with Loss of X Chromosome Inactivation
Source: PLoS One. 2015 Sep 11;10(9):e0137476. doi: 10.1371/journal.pone.0137476 (PMC4567132; doi:10.1371/journal.pone.0137476)

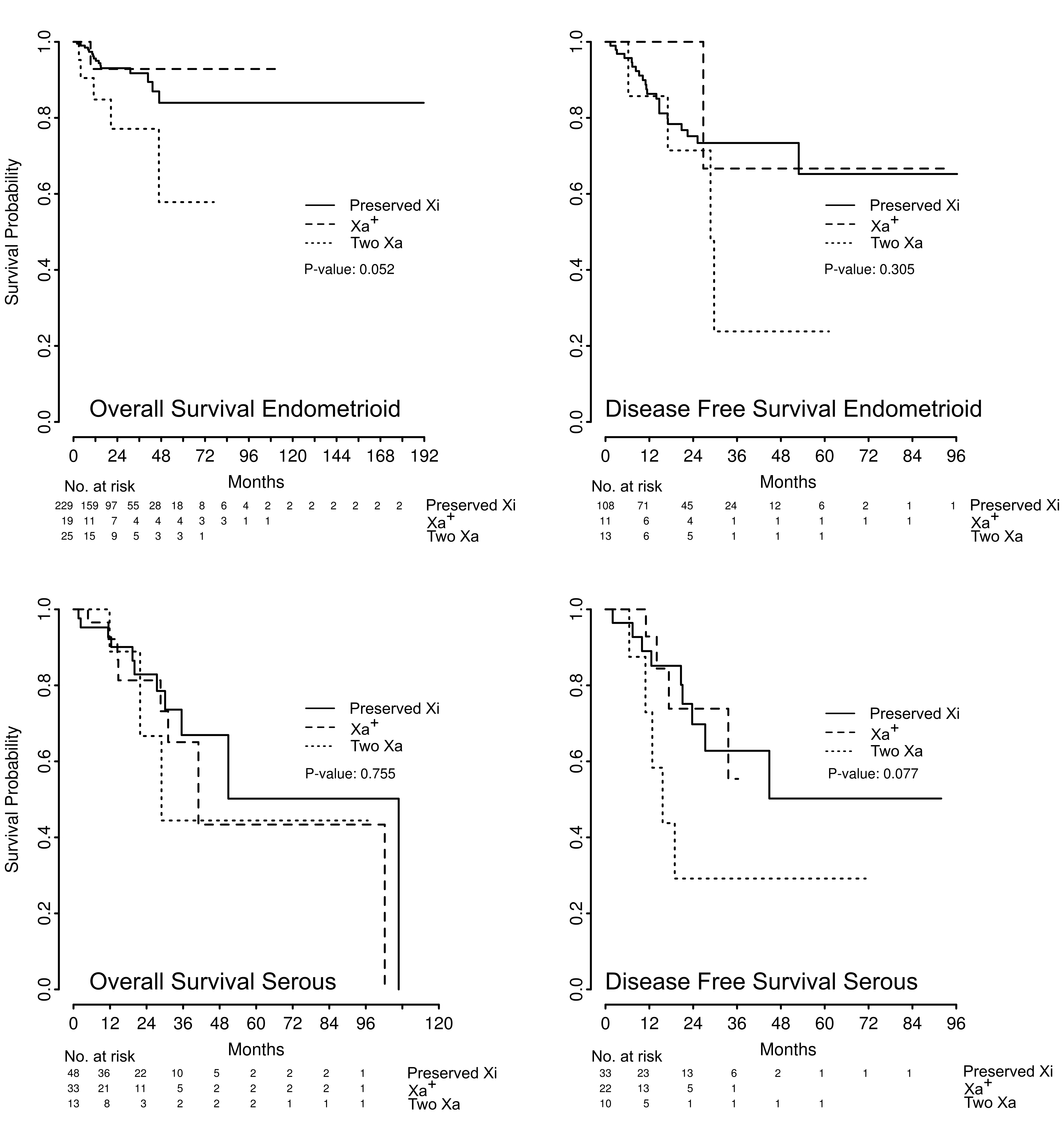

Supplement: S1 Fig — Significance was estimated by the overall log-rank test. (TIF) [file pone.0137476.s002.tif]
