# Supplementary material for: Cancer-Testis Antigen Expression in Serous Endometrial Cancer with Loss of X Chromosome Inactivation
Source: PLoS One. 2015 Sep 11;10(9):e0137476. doi: 10.1371/journal.pone.0137476 (PMC4567132; doi:10.1371/journal.pone.0137476)
Supplement: S1 Table — (DOCX) [file pone.0137476.s003.docx]

**Table S1. Overall and disease free survival data of endometrioid histological type using multivariate Cox regression test**

|  | Overall survival | | Disease free survival | |
| --- | --- | --- | --- | --- |
|  | HR (95%CI) | *P* | HR | *P* |
| Stage I-II | Reference | Reference | Reference | Reference |
| Stage III | 4.15 (1.68-10.28) | 0.002 | 1.63 (0.64-4.21) | 0.308 |
| Stage IV | 4.26 (1.04-17.42) | 0.044 | 5.96 (1.72-20.63) | 0.005 |
| Preserved Xi | Reference | Reference | Reference | Reference |
| Xa^+^ | 0.72 (0.09-5.63) | 0.753 | 0.28 (0.03-2.34) | 0.239 |
| Two Xa | 2.27 (0.76-6.76) | 0.141 | 0.96 (0.27-3.47) | 0.949 |

HR: hazard ratio, CI: confidential interval, Xa^+^: Partial reactivation of Xi, Two Xa: Two copies of Xa

**Table S2. Overall and disease free survival data of serous histological type using multivariate Cox regression test**

|  | Overall survival | | Disease free survival | |
| --- | --- | --- | --- | --- |
|  | HR (95%CI) | *P* | HR | *P* |
| Stage I-II | Reference | Reference | Reference | Reference |
| Stage III | 5.63 (1.65-19.23) | 0.006 | 1.99 (0.47-8.41) | 0.347 |
| Stage IV | 10.89 (3.08-38.53) | <0.001 | 25.96 (5.09-132.51) | <0.001 |
| Preserved Xi | Reference | Reference | Reference | Reference |
| Xa^+^ | 1.64 (0.62-4.35) | 0.319 | 3.62 (0.66-19.95) | 0.14 |
| Two Xa | 1.18 (0.3-4.57) | 0.811 | 8.83 (1.72-45.18) | 0.009 |

HR: hazard ratio, CI: confidential interval, Xa^+^: Partial reactivation of Xi, Two Xa: Two copies of Xa
